# Effects of 10 Hz individualized repetitive transcranial magnetic stimulation on patients with disorders of consciousness: a study protocol for an exploratory double-blind crossover randomized sham-controlled trial

**DOI:** 10.1186/s13063-023-07122-5

**Published:** 2023-04-01

**Authors:** Chengwei Xu, Zhaohua Zhu, Wanchun Wu, Xiaochun Zheng, Haili Zhong, Xiyan Huang, Qiuyou Xie, Xinyi Qian

**Affiliations:** 1 Department of Rehabilitation Medicine, Zhujiang Hospital of Southern Medical University, Guangzhou, Guangdong Province 510280 People’s Republic of China; 2 Clinical Research Center, Zhujiang Hospital of Southern Medical University, Guangzhou, Guangdong Province 510280 People’s Republic of China; 3 School of Rehabilitation Medicine, Gannan Medical University, Ganzhou, Jiangxi province 341000 People’s Republic of China

**Keywords:** Disorders of consciousness, Repetitive transcranial magnetic stimulation, Randomized control trial

## Abstract

**Background:**

Repetitive transcranial magnetic stimulation (rTMS), as a non-invasive brain stimulation technique, has shown potentials for consciousness recovery of patients with disorders of consciousness (DoC), as, to a certain extent, it is effective in regulating the excitability of central nervous system. However, it is difficult to achieve satisfactory effect with “one size fits all” rTMS treatment due to different clinical conditions of patients. There is an urgent need to develop individualized strategy to improve the effectiveness of rTMS on patients with DoC.

**Methods:**

Our protocol is a randomized double-blind sham-controlled crossover trial that includes 30 DoC patients. Each patient will received 20 sessions, in which 10 sessions will be rTMS-active stimulus, and the other 10 sessions will be sham stimulus, separated by no less than 10 days’ washout period. The rTMS-active will include 10 Hz rTMS over the individualized-targeted selection area for each patient according to the different insult regions of the brain. Coma Recovery Scale-Revised (CRS-R) will be used as primary outcome at baseline, after the first stage of stimulation, at the end of the washout period, and after the second stage of stimulation. Secondary outcomes will be measured at the same time, including efficiency, relative spectral power, and functional connectivity of high-density electroencephalograph (EEG). Adverse events will be recorded during the study.

**Discussion:**

rTMS has obtained grade A evidence in treating patients with several central nervous system diseases, and there has been some evidence showing partial improvement on level of consciousness in DoC patients. However, the effectiveness of rTMS in DoC is only 30~36%, mostly due to the non-specific target selection. In this protocol, we present a double-blind crossover randomized sham-controlled trial based on the individualized-targeted selection strategy that aims to study the effectiveness of rTMS therapy for DoC, and the result may provide new insights to non-invasive brain stimulation.

**Trial registration:**

ClinicalTrials.gov: NCT05187000. Registered on January 10, 2022.

## Administrative information

Note: the numbers in curly brackets in this protocol refer to SPIRIT checklist item numbers. The order of the items has been modified to group similar items (see http://www.equator-network.org/reporting-guidelines/spirit-2013-statement-defining-standard-protocol-items-for-clinical-trials/).

**Table:** 

Title {1}	Effects of 10 Hz individualized repetitive transcranial magnetic stimulation on patients with disorders of consciousness: a study protocol for an exploratory double-blind crossover randomized sham-controlled trial
Trial registration {2a and 2b}.	ClinicalTrials.gov: NCT05187000. Registered on January 10, 2022. Statement: all items can be found within the protocol.
Protocol version {3}	November 1, 2021, version 8 (original)
Funding {4}	National Natural Science Foundation of China (No. 82171174, 81974154, 81801119), and Key Realm R&D Program of Guangzhou (No. 202007030005).
Author details {5a}	Chengwei Xu and Zhaohua Zhu contributed equally in this study. They were co-first author.: conceived and designed the study protocol and contributed to drafting the manuscript. CWX and WCW: wrote the manuscript and participated in the coordination and implementation of the study. Xiyan Huang and Qiuyou Xie were both corresponding author.: revised the study protocol and wrote several sections of the manuscript. XCZ, HLZ and XYQ: helped develop the study measures and data collection. All authors contributed to drafting the manuscript and approved the final manuscript.
Name and contact information for the trial sponsor {5b}	Zhujiang Hospital of Southern Medical University. Principal Investigator Dr. Qiuyou Xie, M.D., Ph.D. Address: 253 Industrial Avenue, Haizhu District, Guangzhou city, Guangdong Province, China Email: 764942251@qq.com Tel: +86 13903019604.
Role of sponsor {5c}	This is a researcher-driven study carried out in Zhujiang Hospital of Southern Medical University, with no outside sponsor or funding. The Principal Investigator is actively involved in planning and executing of the study.

## Introduction

### Background and rationale {6a}

Disorders of consciousness (DoC) are a series of arousal and cognitive disorders secondary to severe brain injury [1], encompassing a spectrum of conditions ranging from unresponsive conscious state (UWS) [2] [used to be called vegetative state (VS)] to minimally conscious state (MCS) [3]. Long-term hospitalization of such patients brings up enormous mental suffering and medical burden to their family and society [4]. So far, there is lack of effective treatments for patients with DoC [5].

As one of the non-pharmacological treatments, the neuromodulation technology has been developed rapidly in DoC treatments since 2007 [6]. Repetitive transcranial magnetic stimulation (rTMS) is a form of non-invasive brain stimulation (NIBS) technology which has been recommended to be applied in the treatment of depression, obsessive-compulsive disorder, post-stroke movement disorder and other neurological or psychiatric diseases [7]. Compared to other NIBS, rTMS can be combined with MRI navigation technology to precisely excite or inhibit specific cerebral areas [8] and has a great advantage in exploring connections between different cerebral functional areas [9].

Previously, the application of rTMS in DoC patients was mainly based on the intervention theories related to stroke, such as the interhemispheric competition model, vicariation model, and the bimodal balance-recovery model [10]. Since stroke models mostly focus on brain function reorganization after focal brain injury, it is unknown whether they can cope with the changes in network function caused by extensive brain injury in DoC patients. At present, there are several theories which concern different aspects about neural activity and consciousness. The most prominent maybe Global Workspace Theory (GWT) [11] and Integrated Information Theory (IIT) [12], which highlight different areas such as prefrontal or posterior areas of the brain are crucial for consciousness [13]. According to GWT, dorsal lateral prefrontal cortex (DLPFC) plays an important role in executive control network (ECN), especially in improving mood and cognitive function [14]. Most studies have selected the left DLPFC area as the intervention target and believed that stimulating left DLPFC can strengthen thalamo-cortical and cortico-cortical connections and significantly improve behavioral performance and EEG power spectrum, particularly in MCS patients [15–19]. Meanwhile, according to IIT, the posterior parietal cortex (PPC) is considered as a critical hub in consciousness recovery in default model network (DMN) and also selected as a stimulation target area for DoC patients in some studies [20, 21]. However, until now, the effective rate of rTMS in DoC patients is only 30 to 36% [6]. The most important reason may be that they used the same rTMS stimulation target area for all DoC patients who have different consciousness states and different cortical injury positions.

Therefore, we propose an individualized-targeted selection strategy of rTMS intervention program for DoC patients according to their levels of consciousness and sites of injury. We aim to conduct a randomized double-blind sham-controlled crossover clinical trial that could evaluate the effects of individualized rTMS on DoC patients. The detailed strategies will be described in subsequent sections.

### Objectives {7}

Primary objective

To examine whether10 Hz individualized rTMS is more effective to improve CRS-R scores in DoC patients than the rTMS-sham control

Secondary objective

1. To examine whether 10 Hz individualized rTMS is more efficiency in DoC patients than the rTMS-sham control
2. To examine whether 10 Hz individualized rTMS is more effective to improve relative spectral power of EEG in DoC patients than the rTMS-sham control
3. To examine whether 10 Hz individualized rTMS is more effective to improve functional connectivity of EEG in DoC patients than the rTMS-sham control

### Trial design {8}

**Fig. 1:**
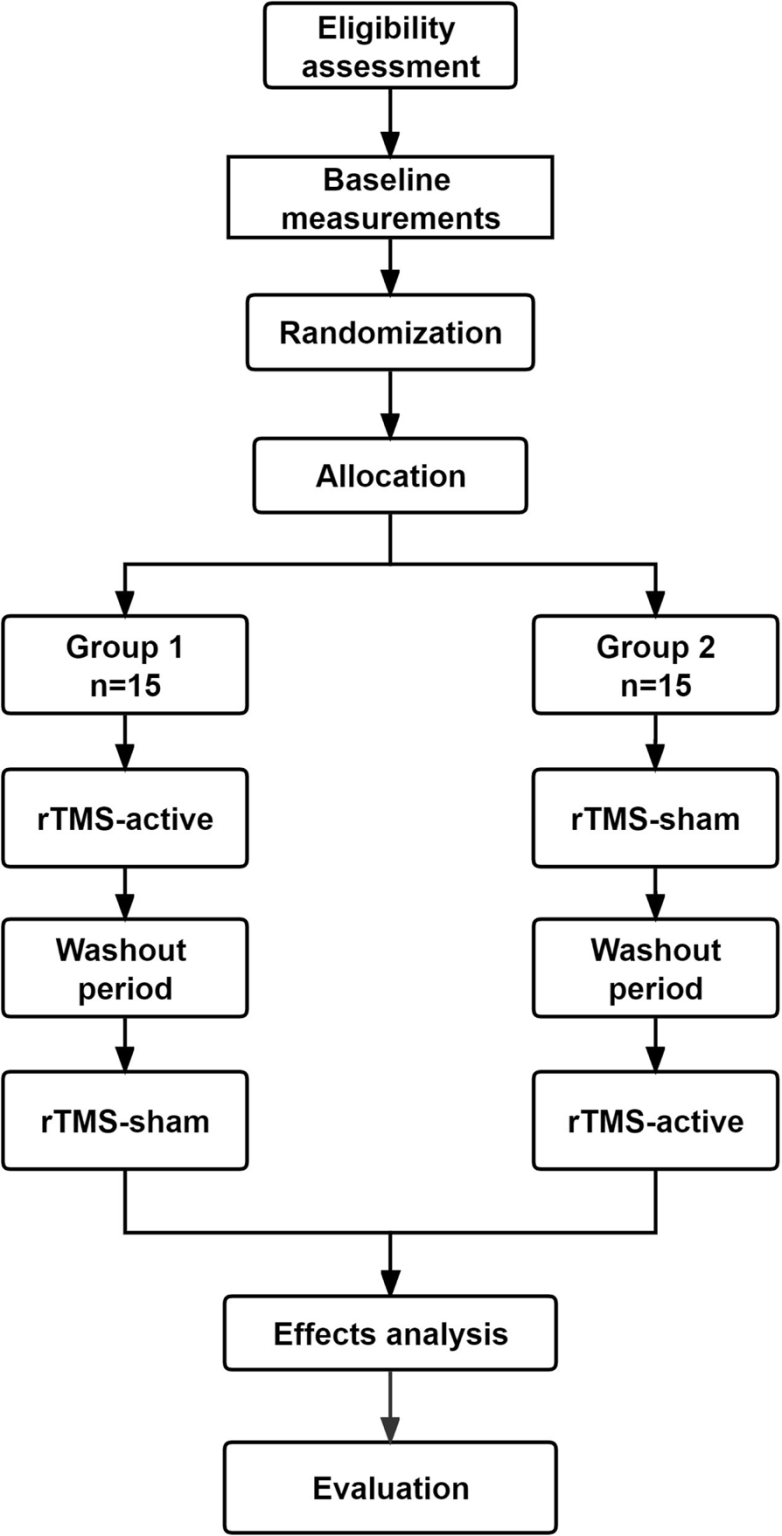
Study protocol flow chart

**Fig. 2:**
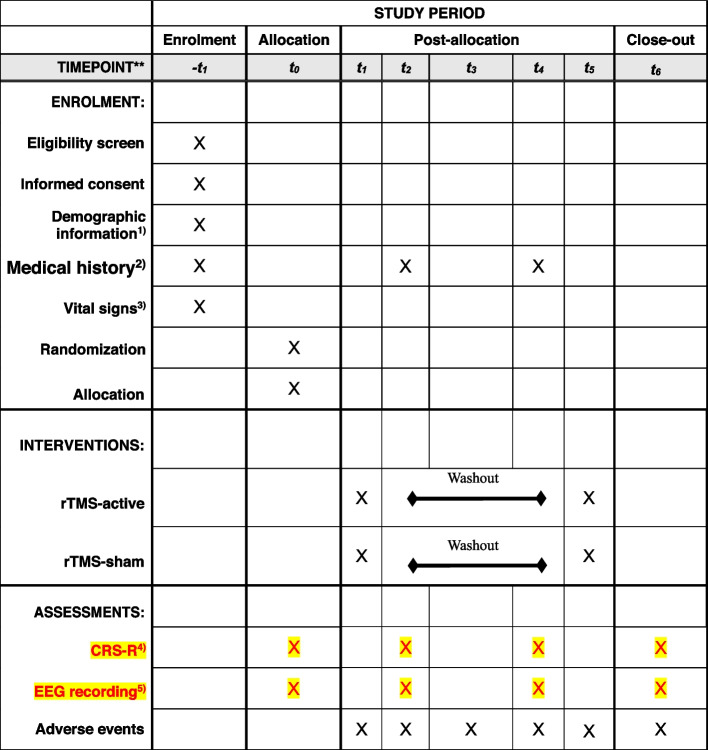
SPIRIT figure. Description of the rTMS study protocol. 1) Demographic information includes age, sex and race. 2) Medical history includes DoC resulting from brain injury and other clinically significant past and present medical history. 3) Vital signs include blood pressure, pulse rate, respiration, and body temperature. 4) CRS-R: Coma Recovery Scale-Revised. 5) EEG, electroencephalogram. 6) rTMS, repetitive transcranial magnetic stimulation. The study protocol was developed according to the guidelines of the Standard Items of the Protocol: Recommendations for Interventional Trials (SPIRIT) guidelines [25]

This study is a crossover randomized, double-blind, sham-controlled clinical superiority trial. Thirty DoC patients will be recruited and divided into two groups in a 1:1 ratio. Group 1 will start with 10 sessions (once a day) of rTMS-active. After no less than 10 days’ washout, this group will be given another 10 sessions (once a day) with rTMS-sham. In contrast, group 2 will do the opposite protocol, participants will start with 10 sessions (once a day) of rTMS-sham, and after no less than 10 days’ washout period will receive 10 sessions (once a day) of rTMS-active. This trial includes a 20-day intervention and a no less than 10 days’ washout period. Five to 10 days washout period has been used in some crossover studies [22, 23] and was shown to be enough to reset the effects [24]. We assume that 10 Hz rTMS applied on the individualized-targeted selection area will improve DoC patients’ level of consciousness. The study protocol flow chart and time of collection of outcomes are described in Figs. 1 and 2, respectively.

## Methods: participants, interventions, and outcomes

### Study setting {9}

The Department of Rehabilitation Medicine and Clinical Research Center (CRC), Zhujiang Hospital of Southern Medical University (SMU), will be responsible for training physiotherapists in a standard operating procedure, and data monitoring committee (DMC) will be responsible for supervising this trial.

### Eligibility criteria {10}

Patients who meet all the following enrollment criteria will enter this study after signing the informed consent: (1) age: 18–70 years old, acquired brain injuries less than 1 year and more than 28 days in DoC; (2) diagnosed as VS/UWS or MCS by CRS-R; (3) no medical history of neuropsychiatric diseases; (4) no sedatives in use or other drugs that might interfere with brain stimulation, such as Na^+^ or Ca^2+^ channel blockers or n-methyl-d aspartate receptor antagonists; (5) stable state of disease and vital signs; (6) the families of the patients volunteer to participate in this study and provided signed informed consent; and (7) the integrity of the individual-targeted selection area are verified by MRI.

Patients who meet the following criteria will be excluded: (1) patients in other non-invasive or invasive neuroregulation trials; (2) motor evoked potential (MEP) in M1 region cannot be induced by TMS pulse; (3) uncontrolled epilepsy, seizure within 4 weeks before enrollment; (4) contraindications for rTMS or EEG, such as metallic implant in the skull, pacemaker, and craniotomy under the stimulated site and implanted brain devices.

### Who will take informed consent? {26a}

Rehab doctors will take informed consent before trial baseline evaluation in the participant reception room. Informed consent will be given by caregivers of potential participants before the time point of allocation.

Informed consent will notify the participants and their caregivers why the study is conducted, what they are going to do, and possible benefits and risks. If the participant has any questions, they are free to ask. The participant’s caregivers can decide whether to participate in the study after fully understanding.

### Additional consent provisions for collection and use of participant data and biological specimens {26b}

This study did not collect additional biological samples. There is currently no subject data available for use in future studies. If related data of participants are needed in the future study, additional consent will be given by participants or their caregivers.

## Interventions

### Explanation for the choice of comparators {6b}

rTMS is a form of NIBS technology in clinic treatment [7]. Compared to other NIBS, rTMS can be combined with MRI technology to precisely excite or inhibit specific cerebral areas [8] and has a great advantage in exploring connections between different cerebral functional areas [9]. In this study, rTMS-sham played a placebo effect in the control group, and there only difference between rTMS-active and rTMS-sham is the pulses whether acting on the cerebral cortex (relevant details are in the “Intervention description {11a}” section is detailed in the section entitled rTMS-sham). In addition, rTMS-sham has been demonstrated in previous studies to be appropriate for the intervention in RCT as a control against rTMS-active [26].

### Intervention description {11a}

#### rTMS

All participants will receive both rTMS-active and rTMS-sham interventions, separated by at least 10-day washout period. Stimulation intensity varies across this experiment and will be determined by the resting motor threshold (RMT) which is defined as the lowest TMS intensity applied to the M1 region. It can evoke electromyography (EMG) with an amplitude > 50 μV peak-to-peak from the right first dorsal interosseous (FDI) muscle at least five out of 10 pulses. If the RMT was above 67%, then the actual intensity of the stimulus was set to 60% of the maximum output of the stimulation device [27]. The researchers will be trained to use the neuronavigation system to mark, and the coil surface will be positioned at a tangent angle of 45° to the scalp [28] on the individualized-targeted selection area to perform rTMS interventions. rTMS pulses will be delivered using an NTK-TMS-II300 stimulator with an IIB502 97-mm figure-of-eight coil. There are two identical surfaces in this coil, one of which can output rTMS-active pluses, and other surface can output rTMS-sham pluses (Brain Modulation Technology Development CO, LDT, JiangXi, CHN). It can produce a biphasic waveform with a pulse width of ~0.32 ms.

#### rTMS-active

During the rTMS-active stage, treatment will be given for 10 consecutive sessions (one session daily). The participants will be placed in the semi-reclining position on either a normal chair or a wheelchair and each stimulation session will last 20 min with a frequency of 10 Hz (train duration: 1s; inter-train interval: 5s; 200 effective stimulation series; 2000 pulses at 90% of RMT). The magnetic stimulation is administered following safety guidelines [27].

#### rTMS-sham

During the rTMS-sham stage, treatment will be given for 10 consecutive sessions (one session daily) also. The sham coil surface has no magnetic field to send to the cerebral cortex while appearing to be the same shape as the active coil, with good approximation of auditory feedback [26]. In our study, the parameters and targeted area of the rTMS-sham are the same as that of the rTMS-active. The only difference is that the rTMS-sham stimulation has no pulse magnetic field sent to the target area but generate noise and vibration.

### Individualized-targeted selection strategy of rTMS for DOC

We propose an individualized-targeted selection strategy of rTMS as the highlight of this protocol. First, two experienced doctors will select a relatively intact brain hemisphere (left or right hemisphere) according to patients’ MRI images. Then, the doctors will select the DLPFC for MCS patients and PPC for VS/UWS patients respectively as their individualized-targeted selection areas. For example, if a patient is VS assessed by CRS-R and his/her left hemisphere is damaged seriously than the right hemisphere, then the right PPC will be selected. Furthermore, if there is no obvious difference between bilateral hemisphere injury, such as hypoxic-ischemic encephalopathy (HIE), and diffuse axonal injury, we will prefer to select the left hemisphere as the stimulation area, because the left DLPFC and left PPC areas have better therapeutic effects in previous studies [16, 18–20, 29].

### Criteria for discontinuing or modifying allocated interventions {11b}

Patients who meet the following criteria will be discontinued intervention: (1) any indication of epilepsy that occurred during the trial and (2) high fever (≥ 38°) more than 3 consecutive days. If the participants who will be excluded as per the criteria will not bring into the trial, and will instead receive all the routine treatment in the Department of Rehabilitation Medicine, we will not collect or use their data.

### Strategies to improve adherence to interventions {11c}

We will provide free rTMS treatment and EEG examination for participants as to improve adherence to interventions.

### Relevant concomitant care permitted or prohibited during the trial {11d}

The relevant concomitant care includes drug therapy (such as amantadine), prevention of complications, and routine rehabilitation treatments. All of the routine rehabilitation programs are provided by qualified rehabilitation therapists from the Department of Rehabilitation Medicine, Zhujiang Hospital of SMU, which include passive limb range-of-motion training, limb electrical stimulation, barometric therapy, respiratory rehabilitation, swallowing therapy, gastrointestinal rehabilitation, and hyperbaric oxygen therapy. All of the above routine rehabilitation treatments will be administered during the trial for all participants. All of other non-invasive or invasive brain stimulation interventions are prohibited (such as transcranial direct current stimulation, transcranial alternating current stimulation, or deep brain stimulation).

### Provisions for post-trial care {30}

Treatment of the participant will be conducted by standard care by rehabilitated protocols after trails. If participants suffer any injury as a result of participating in the trial, we will assess, record, and provide appropriate medical care and pay all relevant medical expenses.

### Outcomes {12}

The primary outcome definition is as follows: domain (disorders of consciousness), specific measure (CRS-R), metric (comparison between active and sham rTMS after intervention), method of aggregation (mean scores of CRS-R), time points (before the experiment (− *t*_1_) and after the end of the first rTMS stage (*t*_2_), after washout period (*t*_4_), and after the second rTMS stage (*t*_6_)). The secondary outcomes are as follows: domain (disorders of consciousness), specific measure (spectral power of EEG and coherence across brain regions), metric (comparison between active and sham rTMS after intervention), method of aggregation (mean proportion of spectral power at five frequency bands and mean coherence), and time points (same as the primary outcome).

### Primary outcomes

#### JFK coma recovery scale-revised

CRS-R [30], as a gold standard, is widely used to define the level of consciousness and assess neurobehavioral recovery in DoC patients. It includes six subscales that assess auditory, visual, motor, motor/speech, communication, and arousal processes. Each item of CRS-R is in good agreement with the diagnostic and differential diagnostic criteria of VS/UWS, MCS, and emergence from minimally conscious state (EMCS) [1]. CRS-R will be recorded at four time points: before the experiment (− *t*_1_) and after the end of the first rTMS stage (*t*_2_), after washout period (*t*_4_), and after the second rTMS stage (*t*_6_). The means of two group levels will be compared.

### Secondary outcomes

#### Efficiency

Based on CRS-R, participant’s level of consciousness from VS/UWS to MCS, from MCS to MCS+, or from MCS+ to EMCS are considered effective. New MCS manifestations in participants with MCS are also considered effective [31] (e.g., patients with autonomous motor responses showed new visual tracking after treatment).

#### Relative spectral power

Relative spectral power will be calculated by the selected artifact-free EEG epochs at five frequency bands: *δ* (1–4 Hz), *θ* (4–8 Hz), *α* (8–13 Hz), *β* (13–30 Hz), and *γ* (30–45 Hz). The relative power of each given band is calculated as follows:

$$RP\left({f}_1,{f}_2\right)=\frac{P\left({f}_{1,}{f}_2\right)}{P\left(1,45\right)}\times 100\%$$

where *P* (*f*_1_, *f*_2_) indicates the absolute power between low *f*_1_ and high *f*_2_ frequency. *P* (1, 45) is the sum of power (1–45 Hz). Then, the relative power for each band was averaged across channels [32].

### Functional connectivity

#### Coherence

For further describing the functional interaction between brain regions, we will perform coherence [33, 34] analysis. Coherence is a measuring method of synchrony of brain activity across different brain regions. We will analyze the coherence of paired channels using 60 channels resting-EEG data, while the values of channel *x* and *y* are calculated by the absolute power spectral density and the cross power spectral density:

$${C}_{xy}(f)=\frac{{\left|{P}_{xy}(f)\right|}^2}{P_{xx(f){P}_{yy}(f)}}$$

where *f* stands for frequency. After obtaining the coherence values of the full frequency band (1–45 Hz), a coherence matrix can be obtained in each brain region by calculating the coherence of the paired electrodes. Then, the coherence values in each frequency band can be obtained by averaging all channel pairs:

$${A}_{xy}=\frac{1}{F_2-{F}_1}{\int}_{F_1}^{F_2}{C}_{xy}(f) df$$

*F*_2_ and *F*_1_ distributions represent the upper and lower frequencies of each frequency band. Finally, the average value of coherence matrix on all data segments is used as the coherence of this frequency band.

The primary outcome is CRS-R, and it will be measured at four time points: before the experiment (− *t*_1_) and after the end of the first rTMS stage (*t*_2_), after washout period (*t*_4_), and after the second rTMS stage (*t*_6_), EEG, as the secondary outcome, will be measured at the same time with CRS-R. The means of two group levels will be compared.

#### Participant timeline {13}

The participant timeline is shown in Fig. 2.

#### Sample size {14}

This is a crossover exploratory study. The CRS-R total score after two-stage treatments will be considered as the primary outcome, while high-density EEG analysis (relative spectral power and functional connectivity) will be the secondary outcome. In this context [35], the sample size calculation will be based on an expected difference between the treatment groups of 20%, setting a significance level of 0.05% and a power of 80%. Considering patients’ compliance and approximately 20% dropout rate during the study, the final sample size of 30 participants is needed. We consider this is an achievable sample size and adequate to allow for the dropout rate while still leaving a reasonable final sample.

#### Recruitment {15}

Participants will be recruited from the Department of Rehabilitation Medicine, Zhujiang Hospital of SMU, with various degrees of DoC [36] caused by traumatic brain injury (TBI) or non-traumatic brain injury (nTBI), including VS/UWS and MCS. Our recruitment advertisement has been posted on the bulletin board of Zhujiang Hospital in November 2021. Patients’ families can contact our principal investigator. After the informed consent is obtained from family members, doctors will conduct screening according to the inclusion and exclusion criteria. Only those who meet all the recruitment requirements and do not meet any exclusion criteria will participate this study.

### Assignment of interventions: allocation

#### Sequence generation {16a}

Participants will be randomly divided into two groups in a 1:1 ratio according to computer-generated randomization using the Random Numbers Function of the statistical software SPSS 23.0 (IBM, USA). Randomization will be performed under the control of a blinded worker from DMC who will be the only person allowed to manage the electronic coding of the randomization to assign the individuals. The researchers will be blind to the group in which the participant is allocated to.

#### Concealment mechanism {16b}

In order to perform the allocation concealment process, the coded groups will be placed in a closed opaque envelope, which will be marked as the code for each participant and held by the staff responsible for randomization. The envelope will only be opened during active or sham rTMS. To ensure proper blinding, participants will be given a code and will be concealed from the allocation process by an independent staff of the DMC who is responsible for randomization. The rTMS experimenter (responsible for applying the intervention) does not know the group allocation either. The rTMS coil will be wrapped in identical opaque plastic papers and labeled A or B. In addition, the rTMS experimenter will be told by one DMC staff (responsible for randomization) to use surface A or B first.

#### Implementation {16c}

The sealed envelopes will be opened and concealed again in the first rTMS stage prior to the intervention by the staff responsible for randomization. Then, the code will inform the rTMS experimenter.

### Assignment of interventions: blinding

#### Who will be blinded {17a}

Both participants and clinic staffs (outcome assessors, caregivers, nurses, physical therapists and statistical analysts, etc.) will remain blind to group allocation. Whether the intervention is rTMS-active or rTMS-sham will not be revealed throughout the study.

#### Procedure for unblinding if needed {17b}

A sealed copy of the cipher will also be prepared for the situation of emergency unblinding. In this study, emergency unblinding can only be implemented for the safety and interests of the participant so that the research intervention (active/sham) given to a participant should be immediately unblinded to determine emergency treatment options. The decision of unblinding must be made by the principal investigator or his authorized representative. The researcher should inform the clinical supervisor of the treatment effects as soon as possible and record the date, time, and reason of unblinding in the original medical record and CRF and then fill out the registration form of emergency unblinding. If serious adverse events occur, the researcher should report serious adverse events within 24 h as required. Once unblinded, the participant will be considered as a shedding case and be withdrawn from the trial, not included in the efficacy analysis, but included in the safety analysis.

### Data collection and management

#### Plans for assessment and collection of outcomes {18a}

All variables in the protocol will be documented in a case report form (CRF). CRS-R and resting-EEG will be collected at the baseline, after the first stage of stimulation, at the end of the washout period, and after the second stage of stimulation. The investigators who enter information into the CRF are responsible for ensuring the accuracy and completeness of information. When the data collection be completed, each CRF will be validated for integrity, consistency, and rationality by the DMC. The DMC will audit through regular interviews or telephone calls.

#### Plans to promote participant retention and complete follow-up {18b}

Not applicable. Study participants will complete all evaluations and treatment during their stay in the hospital.

#### Data management {19}

All data will be handled with utmost care and confidentiality. Data will be stored electronically with passwords and CRF will be stored for the duration of the study and then archived in locked filing cabinets at the Department of Rehabilitation Medicine of Zhujiang Hospital of SMU, for a minimum period of 15 years after the end of the project.

#### Confidentiality {27}

Each participant will be identified by an identification code, including the first letter of the last name and the first letter of the first name. An identification list of participants will be kept in the researcher’s file. Information will be collected for each participant in a CRF filled out by the investigator. Every precaution will be taken to respect the privacy of participants in the conduct of the study.

#### Plans for collection, laboratory evaluation, and storage of biological specimens for genetic or molecular analysis in this trial/future use {33}

Not applicable. No biological specimens will be collected.

### Statistical methods

#### Statistical methods for primary and secondary outcomes {20a}

The SPSS 23.0 statistical software will be used to analysis the results. All the statistical hypotheses will be tested by two-side test, with the statistically significant test level set at 0.05 and the confidence interval estimation of the parameters set to 95%. When the data does not meet the condition of the parameter test, the data transformation can be used; if it still does not meet the condition, the non-parameter test can be considered.

In the descriptive analysis of sample, the baseline means and standard deviation between two groups will be compared for the normally distributed measurement data, and the minimum, maximum, P25, P50, and P75 will be given for the non-normal distributed data. In the final data analysis, firstly, baseline characteristics and carryover effect will be analyzed between the two sequences using independent samples *t*-test and chi-square test. Then, the ANOVA by two stage crossover design will be used if data conform to normal distribution; otherwise, the rank sum test will be used. The above EEG analysis results will take multiple corrections. The enumeration data will be expressed as frequency or percentage, and the chi-square test or Fisher’s exact test will be used to compare the baseline differences between the two groups. If the two stage baselines are different, it means that the crossover design trial has failed. However, clinical data, especially data from studies of DoC patients, are particularly valuable. Assuming this is the case, we will only analyze between baseline and stage1 data.

#### Interim analyses {21b}

No any interim analysis will be conducted.

#### Methods for additional analyses (e.g., subgroup analyses) {20b}

No any additional analysis will be conducted.

#### Methods in analysis to handle protocol non-adherence and any statistical methods to handle missing data {20c}

Both intention-to-treat (ITT) analysis set and per-protocol (PP) analysis set will be used. ITT set includes all participants who have been randomized. When a participant is randomly assigned to an rTMS-active followed by a sham-rTMS, he or she should be included in the rTMS-active followed by a sham analysis. For PP set, only participants who complied with the intervention will be analyzed. In this study, for various reasons, the interruption of rTMS-active no more than 3 days, and the total times of real-stimulation no less than 8 times will all be included in the PP. The ITT analysis will be compared with the PP analysis to determine whether two results are consistent.

#### Plans to give access to the full protocol, participant level-data, and statistical code {31c}

Participant data is sensitive data and cannot be delivered even if name code but will be available for the corresponding author on reasonable request.

### Oversight and monitoring

#### Composition of the coordinating center and trial steering committee {5d}

The Clinical Research Centre (CRC) of Zhujiang Hospital of SMU is an institution which helps to coordinate the work of research team and monitors the clinical trials by setting up the trial steering committee. The trial steering committee consists of a clinical research specialist, a clinical research associate, a clinical research coordinator, and a statistician. It provides supervision over this trial on behalf of the trial sponsor to ensure that the trial is conducted to the rigorous standards set out for good clinical practice.

#### Composition of the data monitoring committee, its role and reporting structure {21a}

This study is administered by the DMC of Zhujiang Hospital of SMU for this single-center study. The DMC consists of specialists in rehabilitation, ethics, statistics, and methodology. The statistician of the DMC checks the data, the ethicist monitors the recruitment, the methodologist and rehabilitation specialist controls the study protocol. The DMC will audit through regular interviews or telephone calls.

#### Adverse event reporting and harms {22}

Possible adverse events and other unintended effects of the trial will be documented on CRF and medical records. The expected hazards in this trial, according to safety guidelines, were epilepsy and hearing impairment, both rated level III. These expected hazards were systematically collected during the screening phase prior to study initiation. If unexpected events occur, we will use MedDRA in the trial publication to report all adverse events that occur. Lethal or severe adverse events will be reported to the Health Commission of Guangdong Province as soon as possible or within 7 days from getting informed of the adverse event.

#### Frequency and plans for auditing trial conduct {23}

Not applicable. No frequency and procedures for auditing trial conduct in this study.

#### Plans for communicating important protocol amendments to relevant parties (e.g., trial participants, ethical committees) {25}

In case of possible future protocol modifications, the Ethical Committee at Zhujiang Hospital of SUM will be informed.

#### Dissemination plans {31a}

The trial results will be published in international peer-reviewed journals focusing on the investigatory field in question.

## Discussion

TMS is a powerful NIBS developed in recent years. The principle is to create a magnetic field energy through the skull, which induces electric field in the cortex to generate an induced current, the membrane depolarization, to modulate the function of key cortical locations [37]. The effect is spread via structural connectivity to other areas of the same network, rebalancing the abnormal activity levels between nodes [38]. Related studies have concluded that rTMS has effects on DoC arousal.rTMS can improve the excitability of cerebral cortical neurons [17] and the functional connectivity of the brain network [23, 39, 40], promote the release of molecules in the brain, and activate regenerative neuropeptide genes such as C-fos and ZIF268 [41]. However, most published trials evaluating TMS as an intervention for patients with DoC had insufficient sample sizes [7]. Moreover, various aetiologies, damaged areas, and injury severity are possible reasons for the unsatisfactory effect of fixed rTMS targets [7, 24]. Previous studies have some drawbacks regarding fixing targets, including selecting a structurally damaged brain area for stimulation that can be avoided. In this study, we aimed to improve the reliability of the existing evidence through a two-stage crossover randomised controlled trial of 30 participants. We hope to validate the existing consciousness models, i.e. GWT and IIT, through the individualised-targeted selection strategy.

To date, the retention/restoration of DMN activity is a fundamental/intrinsic attribute to maintain/enter the function of the MCS [42]. However, the restoration of DMN connectivity alone is not enough to fully restore the consciousness of patients after severe brain injury [43–46]. Since DMN is believed to be involved in mind wandering [47] and self-referential processes [48, 49], it is acceptable that patients with MCS have partially retained self-awareness, daydreaming cognition, or at least residual functional structures [50]. Compared with VS/UWS, their brain network features involve improved DMN but decreased ECN activity [32, 50]. Studies have found significant differences between the MCS and VS/UWS in the left-sided executive control network when comparing the percentage of patients with corresponding independent neuronal activity components [51]. In addition, the difference in hub node sets between MCS and VS/UWS was tested using the index of thresholded connectome intactness, which revealed that patients with MCS retained more normal hub nodes than patients with VS/UWS [52]. It is suggested that these brain areas can be used as candidate targets for non-invasive stimulation to improve patients’ conditions.

The protocol described herein is expected to be the first randomised, controlled, double-blind crossover trial to assess the effects of rTMS intervention in patients with DoC. We hope to demonstrate the effectiveness of rTMS in patients with DoC by prioritising individualised-targeted selection strategies and may also apply this intervention strategy to other NIBS regulations in DoC, such as transcranial direct current stimulation. In our study, CRS-R will be used as a major evaluation index, and EEG will be used as a supplement. The JFK CRS-R estimators are all qualified with unified training. Therefore, this study will provide reliable evidence for the application of individualised rTMS in patients with DoC. We will also actively explore EEG methods for clinical diagnosis and assessment of cognitive function.

## Trial status

The study is currently ongoing at the time of submitting this manuscript (January 2022), using protocol version 8 (1 November 2021). Recruitment started on June 2021, and the study is expected to be completed in December 2022.

## Data Availability

The datasets generated and analyzed during the current study are not publicly available because the protocol has not been completed at the time of submission (see “Trial status” section) but will be available from the corresponding author on reasonable request.

## Abbreviations

DoC: Disorders of consciousness
rTMS: Repetitive transcranial magnetic stimulation
NIBS: Non-invasive brain stimulation
CRS-R: Coma Recovery Scale-Revised
VS: Vegetative state
UWS: Unresponsive wakefulness syndrome
MCS: Minimally conscious state
EMCS: Emerge minimally conscious state
EEG: Electroencephalogram
ICA: Independent component analysis
GWT: Global Workspace Theory
IIT: Integrated Information Theory
DLPFC: Dorsal lateral prefrontal cortex
ECN: Executive control network
DMN: Default mode network
PPC: Posterior parietal cortex
TBI: Traumatic brain injury
nTBI: Non-traumatic brain injury
HIE: Hypoxic-ischemic encephalopathy
MEP: Motor evoked potential
RMT: Resting motor threshold
EMG: Electromyography
FDI: First dorsal interosseous
CRF: Case report form
PP: Per-protocol
ITT: Intention-to-treat
CRC: Clinical Research Center
SMU: Southern Medical University
DMC: Data monitoring committee
